# Performance of Massive Parallel Sequencing-Based Cell-Free DNA Testing in Compromised Pregnancies

**DOI:** 10.3390/jcm13144007

**Published:** 2024-07-09

**Authors:** Maria Antolin, Guillermo Tarrasó, María Ángeles Sánchez, Alberto Plaja, Desiree Martínez-Cruz, Mar Xunclà, Neus Castells, Elena Carreras, Eduardo F. Tizzano, Elena García-Arumí

**Affiliations:** 1 Department of Clinical and Molecular Genetics, Hospital Universitari Vall d’Hebron, Universitat Autònoma de Barcelona (UAB), 08035 Barcelona, Spainelena.garcia@vallhebron.cat (E.G.-A.); 2 Medicine Genetics Group, Vall d’Hebron Research Institute (VHIR), Universitat Autònoma de Barcelona (UAB), 08035 Barcelona, Spain; 3 Maternal-Fetal Medicine Unit, Department of Obstetrics, Hospital Universitari Vall d’Hebron, Universitat Autònoma de Barcelona (UAB), 08035 Barcelona, Spain; 4 Research Group on Neuromuscular and Mitochondrial Disorders, Vall d’Hebron Research Institut (VHIR), Universitat Autònoma de Barcelona (UAB), 08035 Barcelona, Spain; 5 Centro de Investigación Biomédica en Red de Enfermedades Raras (CIBERER), Instituto de Salud Carlos III, 08041 Barcelona, Spain

**Keywords:** cell free fetal DNA analysis, NIPT, GWNIPT, fetal ultrasound abnormality, early pregnancy loss

## Abstract

**Background/Objectives**: Non-Invasive prenatal test (NIPT) is used as a universal or contingent test after prior risk assessment. Screening is mainly performed for common trisomies (T21, T13, T18), although other chromosomal anomalies may be detected. Our objective was to study the performance of GWNIPT in the detection of chromosomal abnormalities in pregnancies in which an invasive prenatal study was performed and in early pregnancy losses, in comparison with the reference test. **Method:** VeriSeqTM NIPT Solution v2, a genome-wide NIPT (GWNIPT), was performed prior to invasive testing in fetal diagnostic study cases (FDS, *n* = 155) and in early pregnancy losses (EPL, *n* = 68). **Results:** In the FDS group, the diagnostic test (QFPCR, array and karyotype) detected anomalies in 32 pregnancies (21%), in twenty of them (61%) also detected by GWNIPT. Eleven of the twelve cases undetected by GWNIPT were balanced translocations (*n* = 4) or deletions/duplications <7 Mb (*n* = 7). In the EPL group, GWNIPT detected anomalies in 46% of cases (31/68) but comparison with reference test (QFPCR and karyotype) in products of conception (POC) was only possible in 18 cases. Concordant results between POC and GWNIPT test were obtained in 16 of the 18 cases. In EPL, with GWNIPT testing, common trisomies accounted for 25.8% of cases (8/31), rare trisomies 54.8% (17/31) and microdeletions/duplications 16.1% (5/31). **Conclusions**: The GWNIPT test may be useful in clinical practice in prenatal and in EPL’s genetic diagnosis when the appropriate sample is not available.

## 1. Introduction

Non-Invasive prenatal test (NIPT), based on cell-free fetal DNA (cffDNA) circulating in maternal plasma, was introduced in clinical practice in 2008 [1]. Since then, the number of countries implementing this technology as a national screening strategy has been growing. NIPT is being used either universally or in a high-risk population, such as after the first-trimester combined test [biochemical parameters fβ-hCG (free beta-human chorionic gonadotropin), PAPP-A (Pregnancy associated plasma protein-A) and nuchal translucency] to decrease the need for invasive testing. In both strategies, universal or contingency screening, NIPT is mainly used for the most common trisomies, T21, T18 and T13 [2,3,4] and has been useful in reducing the number of invasive tests, although the possibility of discordant results due to chromosomal mosaicism is not excluded [5,6]. This is the main reason why NIPT is now considered a neonatal screening test that should be confirmed by an invasive reference test whenever possible.

While T21, T18 and T13 are the most prevalent, many other chromosomal alterations may cause early fetal malformations or early pregnancy loss. Genome-wide approaches to NIPT (GWNIPT) could potentially allow the detection of other chromosome alterations [7], but their performance remains challenging and controversial. With arguments for and against, genome-wide versus targeted testing is still under debate [8]. Although universal GWNIPT can increase sensitivity in the detection of genetic abnormalities and achieve a high degree of satisfaction in pregnant women, the risks of discordant positives, with increased invasive diagnostic testing and the parental anxiety that may result, are major drawbacks when considering its implementation [9,10]. However, some evidence has been found on the clinical impact of screening for additional findings [11,12].

When a fetal diagnostic study (FDS) is necessary due to fetal ultrasound abnormalities (FUA), high-risk combined screening or an abnormal finding in a previous gestation, invasive tests like chorionic villus sampling or amniocentesis are offered to allow the detection of chromosomal anomalies [13] using quantitative fluorescence polymerase chain reaction (QFPCR), karyotype, microarray analysis and, recently, CNVseq [14]. Exome sequencing in invasive samples is also an option to take into account in prenatal diagnosis to detect pathogenic SNV, indels or small CNV below microarray resolution [15]. Sometimes, in situations such as oligohydramnios (4.4% of gestations) [16], renal anhydramnios in early pregnancy (1/2000 gestations) [8] or refusal of invasive testing, GWNIPT could be an alternative.

Early pregnancy loss (EPL) is defined as a nonviable, intrauterine pregnancy with either an empty gestational sac or a gestational sac containing an embryo or fetus without fetal heart activity within the first 12 6/7 weeks of gestation. One in ten pregnancies ends in EPL, and approximately 50% of all cases are due to chromosomal anomalies [17]. QFPCR, karyotype and microarray analyses of the products of conception (POC) are the gold standard to determine whether this is the cause. Different situations may make these tests unfeasible, such as POC not being available because the loss occurred outside the hospital, or maternal contamination. The GWNIPT test could be used as a “second best” in these circumstances.

The aim of this study was to evaluate the performance of a GWNIPT test (VeriSeqTM NIPT Solution v2, Illumina, San Diego, CA, USA) for the detection of fetal chromosomal anomalies in cohorts of compromised pregnancies as fetal abnormalities and early pregnancy loss.

## 2. Materials and Methods

This study was performed between March 2019 and February 2021 at the Department of Maternal-Fetal Medicine in collaboration with the Department of Clinical and Molecular Genetics of the Vall d’Hebron University Hospital in Barcelona, Spain. The study was conducted according to the guidelines of the Declaration of Helsinki, and approved by the Ethics Committee of the Vall d’Hebron University Hospital, Barcelona, Spain (PR(AG)529/2018 approval in 22 February 2019).

The study population included two different groups, which were recruited prospectively. Group A (FDS) included 155 pregnant women who required a fetal diagnostic study due to FUA, high risk after combined screening in the first trimester, parental genetic anomaly or previous pregnancy anomaly (Table 1); for this purpose, they underwent invasive tests (amniocentesis or chorionic villus sampling). Group B (EPL) included 68 women, who were seen in the Emergency Department of our hospital, with ultrasonographically confirmed EPL and with the conceptus still in the uterus. The participating couples received pre- and post-genetic counseling following the standard protocol of Vall d’Hebron Hospital. In addition, all participants gave their informed consent to be included in the study and for their data to be used anonymously for research purposes.

All participating women underwent a blood draw for cfDNA analysis. In group A, the blood sample was obtained prior to invasive testing in the Obstetrics Department. In group B, the blood sample was collected in the Emergency Department of our hospital, after ultrasound confirmation and before uterine curettage or drug treatment for expulsion. DNA was extracted from uncultured or cultured samples of amniotic fluid (AF), chorionic villus (CV) biopsies or products of conception (POC) using the iGENatal genomic DNA extraction kit (genBiotech, Madrid, Spain), according to the supplier’s recommendations.

In the FDS group, DNA from AF, CV or POC was first analyzed with the QF-PCR Devyser Complete kit (QFPCR, aneuploidy analysis of chromosomes 13, 18, 21, X and Y; Devyser, Stockholm, Sweden) following the manufacturer’s protocol. If no anomaly was detected, the CGH array was carried out. If abnormal QFPCR results were obtained, they were confirmed by karyotype analysis (resolution of 400–550 bands with a detection limit of 5–10 Mb). In addition, the rare trisomies and some duplication/deletion larger than 5–10 Mb detected by microarray were confirmed by karyotyping. Finally, karyotype studies were also performed in all cases with family suspicion of balanced translocations or derivatives. The CGH array (ogt 020045, CytoSure Constitutional v3, 8 × 60 K, OGT, UK (https://www.ogt.com/products/product-search/cytosure-constitutional-v3-and-v3-loh-arrays/ (accessed on 9 May 2024)) had a resolution average of 663 Kb and of 189–375 Kb in the regions defined by the ISCA consortium (International Standard Cytogenetic Array), and an exon resolution in 354 genes associated with developmental delay identified by ClinGen (National Institutes of Health, Bethesda, MD, USA).

In the EPL group, DNA from POC was analyzed with the QF-PCR Devyser Extend v2 kit (QFPCRE, aneuploidy analysis of chromosomes 13, 15, 16, 18, 21, 22, X and Y; Devyser, Stockholm, Sweden,). The detected aneuploidies were confirmed by karyotype analysis when POC cell culture was available. Additionally, after normal QFPCRE results, karyotyping and/or microarray was performed (on fresh or cultured sample, when available).

Cell-free fetal DNA (cffDNA) testing was performed in both groups with whole genome sequencing analysis, using VeriSeqTM NIPT Solution v2 (Illumina) with standard LLR (likelihood ratio value) thresholds. Test menu options allowed for the analysis of common autosomal aneuploidies (CAAs, chromosomes 21, 18 and 13), all rare autosomal aneuploidies (RAAs), sex chromosome aneuploidies (SCAs) and partial deletions and duplications (CNVs) of at least 7 Mb in size. The workflow of the analysis included cfDNA isolation from maternal blood, library preparation, next-generation sequencing in a NextSeq 550, data analysis and interpretation.

In all the pregnancies, the following data were collected: maternal and gestational age, type of invasive test (CV, AF and POC), QFPCR/QFPCRE/array/karyotype and GWNIPT results, and follow-up of cases with detected chromosomal anomaly. The results of GWNIPT were not communicated to the participants.

## 3. Results

### 3.1. Group A: Fetal Diagnostic Study (FDS)

**Figure 1:**
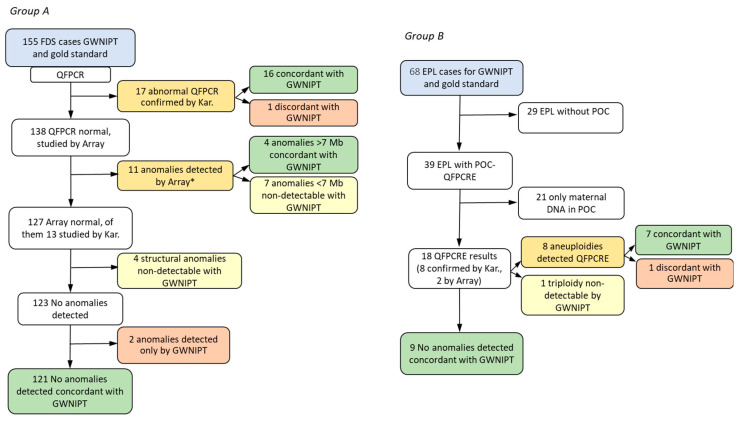
Flow chart describing the diagnostic algorithm and comparison of results obtained with GWNIPT and gold standard in group (**A**) (Fetal Diagnostic Study, *n* = 155) and group (**B**) (Early Pregnancy Loss *n* = 68). * 1 case with another anomaly <7 Mb not detectable by GWNIPT. In orange, anomalies detected by the gold standard; in green, concordant anomalies gold standard/GWNIPT; in red, discordant anomalies gold standard/GWNIPT; in yellow, non-detectable anomalies by GWNIPT.

In the FDS group, which included 155 women with indication for invasive tests, chorionic villus biopsy was performed in 96, amniocentesis in 57, cordocentesis for fetal blood sampling in 1, and POC remains were analyzed in 1 case. The gestational age at invasive testing was between 11 + 2 and 29 + 2 weeks + days (mean 15.3 weeks, median 13.7). The array study was performed on all samples, excluding 17 cases in which abnormalities had previously been detected by QFPCR and confirmed by karyotyping (7 cases of T21, 5 T13, 3 T18 and 2 X0) (Figure 1 and Table 2).

Following the diagnostic algorithm shown in Figure 1A, 32 cases with genomic anomalies were detected in the FDS group (21% of the pregnant women) using the reference diagnostic test (QF-PCR/array/karyotype) [18,19]; 20 of them were also detected using GWNIPT (62.5%, 95% CI: 42.5–77.1). In one case, where the array detected two CNVs, dup(12)(p13.33p11.1) and del(18)(p11.32), GWNIPT only detected the first (Table 2).

As can be seen in Table 2, most of the FUA were associated with pathogenic chromosomal anomalies detected with the gold standard. The exceptions were one case with FGR and deletion del(12)(q12) of 0.81Mb (includes the gene *PDZRN4*) of still uncertain significance; and two cases of FUA, a T8 and a T16 detected by GWNIPT and not detected by the reference methods (array or karyotype) in samples of amniotic fluid (Figure 1A, Table 2). There were 12 cases with anomalies not detected by GWNIPT: 4 were balanced translocations (one pericentric inversion and 3 translocations) with no relevance in the context of ultrasound anomalies, 7 cases (8 anomalies) were expected discordances due to the size [Table 2, 4 small duplications <1.5 Mb and 4 small deletions <2.5 Mb], and 1 case of mosaic Turner. In this case, with a fetal fraction estimate (FFE) of 4%, the normalized X value was outside the XX region, but did not raise the X0 zone. The detection rate of GWNIPT in relation to abnormalities detected by fetal ultrasound was 22/28, 78.6%; while for the gold standard it was 25/28, 92.6%”.

In summary, taking into account its current limitations, GWNIPT correctly detected 20 of the 21 detectable cases, which represented 95.2% (95% CI: 77.3–99.8%). Eleven of the 32 cases with anomalies in the FDS group were outside the detection range of the GWNIPT test, representing 34.4%. Therefore, in our study, 65.6% of all FDS cases with chromosomal abnormalities could have been detected by a noninvasive procedure.

### 3.2. Group B: Early Pregnancy Loss

In group B, 68 women with EPL confirmed by ultrasound were recruited. Gestational age at blood draw, which was recorded in 59/68 cases, was 8.6 ± 1.9 weeks (mean ± SD). After processing the POC samples, it was only possible to analyze 18 of the 68 cases. (Figure 1B). However, GWNIPT test results were possible in all cases, despite the fact that most blood samples were obtained before 10 weeks of gestation.

In POC samples, chromosomal anomalies were detected in 9 of the 18 cases (50%), 8 trisomies and 1 triploidy (Figure 1, Table 3). T21 was the most frequent anomaly (4/9, 44%), whereas RAAs represented together 33% (1 T15, 2 T22). GWNIPT detected 7 of the anomalies (7/8, 87.5%), whereas one T22 was undetected (in a sample with 2% of FFE). In the other 9 cases, no chromosomal anomalies were detected with either the reference method or GWNIPT.

In the remaining 50 cases of EPL, POC results comparison was not feasible (Figure 1B, Table 4). In 29 of them, EPL occurred outside the hospital or a POC sample was not obtained during emergency care, and in 21 cases POC was collected but was exclusively of maternal origin. Gestational age was lower in maternally contaminated samples (7.8 ± 1.9 vs. 10.9 ± 1.8 weeks, mean ± SD *p* < 0.05). Low gestational age could be associated with difficulty in obtaining adequate POC for the study. In these 50 cases, GWNIPT detected chromosomal anomalies in 24 of them (48%) (Table 4): 18 cases of trisomy, 2 of monosomy, 1 deletion and 4 cases of duplication (one case had duplication and T16). T21 represented in this subgroup 12.5% (3/24) of the anomalies detected, whereas RAAs (T4, T6, T9, T14, T15, T16, T20, and T22) accounted for 62.5% (15/24).

Overall, in the entire EPL group, GWNIPT detected chromosomal anomalies in 31 of the 68 cases, representing 46.3% of the group. T21 was also the most frequent anomaly (23%, 7/31). Gestational age was significantly higher (*p* = 0.019) in T21 cases than in the whole EPL group (10.7 ± 1.9 vs. 8.6 ± 1.9 weeks, mean ± SD). In addition, T21 cases showed higher FFE (9.3 ± 4.4%) than cases with other anomalies (5.6 ± 3.0%). The FFE in the EPL group was 6.6 ± 4.0, range 1–17. RAAs accounted for a high proportion of cases with anomalies detected by GWNIPT, 54.8% (17/31), while CAAs were 25.8% (8/31) and CNVs 16.1% (5/31), data comparable to those obtained in the POC-confirmed subgroup.

## 4. Discussion

Fetal malformation and early pregnancy loss are frequently due to numerical or structural chromosome anomalies. Detection of these anomalies can be relevant for clinical management of compromised pregnancies and counseling. Invasive techniques or analysis of products of conception are usually performed for this purpose, but are not always feasible or desired. GWNIPT could, to a certain extent, fill the gap in these situations.

In our FDS, most of the ultrasound anomalies were associated with pathogenic chromosomal anomalies detected with the gold standard. Overall, a good correlation between GWNIPT and cytogenetic analysis was also found in our FDS study. Among the 32 cases detected by cytogenetic analysis, 63% (20/32) were also detected by GWNIPT. Four undetected cases were hereditary balanced translocations detected by karyotyping that were tested in the fetus for unbalanced congenital chromosomal abnormalities, and which were irrelevant in the context of the ultrasound abnormalities. Seven non-detected cases were below the detection limit of GWNIPT, five of them considered pathogenic. In the case of the 0.29Mb duplication (dup(X)(q28), that leads to *MECP2* gene duplication syndrome [20], the mother was a carrier. Its inheritance is X-linked recessive, with affected male carriers (100% penetrance). Lastly, one case of Turner mosaic was not detected by GWNIPT despite the fact that the percentage of mosaic was approximately 70% in the chorionic villi. In the first trimester ultrasound, this fetus presented anomalies such as generalized mild skin edema and suspected unilateral mild hydrothorax, corresponding to the detected anomaly. The result was not confirmed in amniotic fluid, as it was considered fully concordant. Additional efforts are needed to improve the sensitivity in detecting mosaics on the X chromosome.

In two additional cases, T16 and T8 were detected by GWNIPT but not by array/karyotyping in amniotic fluid. Both discrepancies could be attributed to confined placental mosaicism (CPM). In the T16 fetus, fetal growth restriction (FGR) was observed, which could be associated with a T16 confined placental mosaicism, as previously reported in CV and AF samples in other studies [21,22]. Unfortunately, confirmatory placental studies were not possible. GWNIPT could help diagnose a placental-limited genetic abnormality as a cause of FGR. Future studies should be performed to address this point. The implementation of universal GWNIPT is currently under debate [7,8,9,10,11,12]; however, in compromised pregnancies, as in our FDS group, the percentage of cases with rare anomalies was considerable, 11% (17/155), representing approximately half of all detected anomalies (32/155, 20.6%), and some of them (5/17) were detectable by GWNIPT (Table 2). Previous prenatal studies found a comparable percentage of abnormalities by karyotyping (10.6%, [23]) or microarray (15%, *n* = 89, [24]) with a similar distribution [23] in CAAs (7.4% vs. 9.6% in this study), SCAs (1.3% vs. 1.2%), RAAs (0.1% vs. 0.6%) and CNVs (6.0% vs. 6.4% by array, only 1.9% detected by GWNIPT). The difference in RAA occurrence is probably due to the small sample size of our cohort. Another study of 89 FUA gestations found 12% CNVs by array [24], and approximately half were of detectable size by GWNIPT. However, in our study, only one-third of CNVs reached this size. The different resolution of the arrays and the relatively small number of cases in both studies could help to explain these variations.

In summary, when prenatal genomic studies are needed, invasive testing followed by microarray analysis or whole exome sequencing is the first choice. When amniocentesis is impossible due to lack of amniotic fluid, or is refused by the pregnant woman, GWNIPT is a potential alternative. This study achieved a detection rate of 95.2% (CI: 77.3–99.8%) for anomalies detectable by GWNIPT, 71% (CI: 52.9–84.8%) if anomalies <7 Mb (detected by array) are included, and 62.5% (CI: 45.3–77.1%) if balanced structural variants (detected by karyotyping) are also included. Further improvements to the coverage and detection algorithm should be introduced to increase the sensitivity of CNVs in GWNIPT. Deeper sequencing is a very relevant factor for the detection of smaller anomalies, although it has the disadvantage of increased costs. In addition, the use of positive controls to improve the detection algorithm can optimize the increase in sensitivity. To address this point, a balance between cost and benefit must be reached.

The EPL guidelines recommend cytogenetic analysis of POC in third and subsequent pregnancy losses [25,26], but, in clinical practice, POC are often unavailable or too contaminated with maternal tissue for reliable analysis. Cell-free DNA analysis of the entire genome of maternal blood may be an alternative technique, but to date, few studies have evaluated its potential usefulness. In this study, GWNIPT detected chromosomal anomaly in 46.3% of the first trimester miscarriages. This result agreed with the 50% of anomalies found in our 18 EPL cases with POC results. Moreover, comparable data were obtained in studies performed in POC (70.3% [27], 47.3% [28], 60% [29], 61% [30]) or in maternal blood (GWNIPT) (50% [29], 55% [31], 28% [32] 24% [33]). Interestingly, the percentage of chromosomal anomalies was similar to that obtained by Yaron (55%, [31]) and higher than that of Colley (28% [17]) using the same GWNIPT methodology. Given that the number of participants was similar (Yaron *n* = 109, Colley *n* = 57, this study *n* = 68), the differences may be partly explained by the small size of these cohorts.

Previous research found GWNIPT to be more sensitive for chromosome anomalies restricted specifically to the placenta than chorionic villus sampling [33]. In this study, the QFPCR/karyotype results are only representative of the analyzed piece of POC, and consequently, the anomaly might not be present in the sampled region. However, in our comparative study, we found a good correlation between the results obtained by the GWNIPT test and the results of the POC analysis using standard methods. Sixteen of the 18 samples with POC results had results concordant with GWNIPT (7 with the same anomaly and 9 euploid) and no additional cases of chromosomal anomalies were found by GWNIPT. However, a T22 case was only detected by QFPCR/karyotype in POC, whereas it was close but below the threshold in GWNIPT. The relatively low FFE of this sample (2%) could have contributed to this discordant result.

The anomalies found by GWNIPT in the EPL group (T21 23% of abnormalities detected, T15 11%, T16 13%, T20 8%, T22 11% and X0 11%), have previously been associated with miscarriage [34]. The proportion of T21 cases was higher than in other studies (9.1% [34], 7.4% [17], 3.6% [31]), possibly due to their high gestational age and FFE [34]. The CNVs detected by GWNIPT in EPL are large enough to be pathogenic. In addition, according to the guides of the of the American College of Medical Genetics [18,19], all of them can be associated with miscarriage except dup(12)(q23.1q24.32). Moreover, the detection rate of abnormalities by GWNIPT in the whole group (46%), as well as in the subgroup with POC comparison (50%), was similar to that reported by others, making our findings plausible.

Confirmation of a chromosomal anomaly in case of EPL is a relief for the pregnant woman, as it rules out other external factors, and improves the chances of success in future pregnancies. Our study and others confirm the possibility of using GWNIPT in EPL, even before week 10 of pregnancy, with informative results of chromosomal anomaly. We speculate that sampling in the Emergency Department, at the time women are diagnosed with EPL, is the key.

Our study highlights not only the difficulty of obtaining POC, but also that these POC were not contaminated with maternal content. In this study, adequate POC samples were obtained in only 18 of 68 cases of EPL (26.5%) whereas GWNIPT samples with evaluable results were obtained in all cases. In our hospital, approximately 90%, or even more, of women with EPL arrive at the Emergency Department with the conceptus still in the uterus, which means that they can be tested by GWNIPT. These facts underline the relevance of the GWNIPT test in these situations, as it allows relevant information to be obtained for future pregnancies in these women.

## 5. Conclusions

The use of cell-free fetal DNA-based approaches to detect chromosomal anomalies in fetuses with ultrasound abnormalities or high-risk combined screening, or in early miscarriages, may be useful in clinical practice when the reference methodology is not available due to the specific patient situation or technical problems. In pregnant women with FUA or high-risk combined screening who refuse prenatal invasive testing, or in women with oligohydramnios with no invasive testing possible, GWNIPT has clinical utility in detecting fetal chromosomal abnormalities. In cases of EPL, when POC is unavailable or of inadequate quality, GWNIPT may play an important role as a reliable alternative technique, although of lower sensitivity.

## Figures and Tables

**Table 1:** Clinical characteristics of pregnant women included in the Fetal Diagnostic Study (*n* = 155).

Indication for Prenatal Diagnosis	*n* Cases	%
Fetal Ultrasound Anomalies	69	44.5
Multiple	13	8.3
Central Nervous System	7	4.5
Cardiac	8	5.1
Urogenital	5	3.2
Skeletal	2	1.3
Gastrointestinal	2	1.3
Liver	1	0.6
Nuchal Translucency	23	14.8
Fetal Growth Restriction	7	4.5
Hydrops	1	0.6
High Risk Combined Screening	43	27.6
Progenitor genomic anomaly *	22	14.2
Antecedent Previous Gestation	19	12.2
Other ^#^	2	1.3

* Structural variant (*n* = 9) or molecular pathogenic variant (*n* = 13). ^#^ chemotherapy, toxoplasmosis.

**Table 2:** Chromosomal anomalies found in the Fetal Diagnostic Study group (FDS, *n* = 155). QFPCR or array and/or karyotype detected 32 cases of anomalies in these pregnancies, whereas GWNIPT detected 22 cases (2 of them not detected by QFPCR, array or karyotype).

Chromosomal Anomaly	Sample	QF-Kar-Array Detection ^&^	GWNIPT Detection	Size (Mb)	Boundaries [GRCh37] ^$^	Pathogenicity ^ϒ^	FUA
T7	CV	Array	Yes			Pat	FGR
T13 (*n* = 5)	CV 3/AF 2	QF-Kar ^#^	Yes			Pat	MFA, OA, HF
T18 (*n* = 3)	CV	QF-Kar ^#^	Yes			Pat	NT, CA
T21 (*n* = 7)	CV 6/AF 1	QF-Kar ^#^	Yes			Pat	NT, CA, HF
45,X	CV	QF-Kar ^#^	Yes			Pat	CH
dup(9)(p24.3p13.1)	CV	Array- Kar	Yes	38.8	9p24.3p13.1(204090_38815471)x3~4	Pat	CH, KA
dup(12)(p13.33p11.1)/ del(18)(p11.32) *	CV	Array- Kar	Yes/No	33.7/1.6	12p13.33 p11.1(244335_33986995)x3, 18p11.32(149089_1754474)x1	Pat, VUS	NT, CA, FGR
del(1)(q43q44)	AF	Array	Yes	7.9	1q43 q44(241293508_249203359)x1	Pat	FGR, CNS
dup(1)(q21.1q21.2)	CV	Array	No	2.6	1q21.1q21.2(145899359_148545664)x3	Pat	CNS
dup(9)(p24.3)	AF	Array	No	0.45	9p24.3(343893_789747)x3	Pat	CNS
mos dup(X)(q25)	CV	Array	No	0.78	Xq25(122869563_123646637)x2~3	VUS	Non-detected
dup(X)(q28) **	CV	Array	No	0.29	Xq28(153120541_153406100)x2	Pat in XY	KA
del(12)(q12)	AF	Array	No	0.81	12q12(41634139_42447650)x1	VUS	FGR
del(15)(q25.2q25.3)	CV	Array	No	2.4	15q25.2q25.3(83283395_85666184)x1	Pat	NT
del(X)(p21.1) **	AF	Array	No	0.42	Xp21.1(31787544_32205055)x0	Pat in XY	Non-detected
t(5;12)(p10;q10)mat	CV	Kar	No			No, maternal	Non-detected
t(6;11)(q24;q12)mat	CV	Kar	No			No, maternal	Non-detected
der(13;14)(q10;q10)pat	CV	Kar	No			No, paternal	Non-detected
inv(7)(p22q32)pat	POC	Kar	No			No, paternal	Non-detected
mos 45,X	CV	QF-Kar ^#^	No			Pat	HF
T8	AF	No	Yes			Pat	CNS
T16	AF	No	Yes			Pat	FGR

CV, chorionic villi; AF, amniotic fluid; POC, products of conception; QF, QFPCR; Kar, karyotype; Array, microarray; Pat, pathogenic. FUA, Fetal Ultrasound Anomaly; FGR, Fetal Growth restriction; MFA, Multiple fetal ultrasound anomalies; OA, Oligohydramnios; HF, Hydrops fetalis; NT, nuchal translucency; CA, Cardiac anomalies; CH, Cystic Hygroma; KA, Kidney anomalies; CNS, Central Nervous System. ^&^ Test that detected the anomaly. ^#^ As anomaly was detected by QFPCR and confirmed by karyotype, microarray was not performed. * Fetus with unbalanced translocation 46,XX,der(18),t(12;18)(p11.1;p11.32 whose father was a carrier of a balanced translocation between chromosomes 12 and 18. [46,XY,t(12;18)(p11.1;p11.32)]. ** fetus XY. ^$^ Boundaries of CNVs determined by microarray. ^ϒ^ According to the guides of the American College of Medical Genetics [18,19].

**Table 3:** Chromosomal anomalies found in EPL detected by the gold standard (QFPCR-Expanded/Karyotype) and GWNIPT (*n* = 18 cases). In the remaining 9 cases no chromosomal anomalies were detected.

Patient	QFPCRE-Kar Study	QFPCRE-Kar Result	GWNIPT Result	GA wks	FFE %
1	QFPCRE	T15	T15	8	5
2	QFPCRE	T18	T18	12	2
3	QFPCRE, Kar	T21	T21	9	8
4	QFPCRE, Kar	T21	T21	13	8
5	QFPCRE	T21	T21	12	11
6	QFPCRE	T21	T21	13	9
7	QFPCRE	T22	T22	11	9
8	QFPCRE, Kar	T22	NAD	10	2
9	QFPCRE, Kar	Triploid 69, XXY	NAD	9	12

QFPCRE, QFPCR-Expanded; Kar, karyotype; NAD, no anomaly detected; GA, gestational age; wks, weeks; FFE, fetal fraction estimate.

**Table 4:** Chromosomal anomalies detected by GWNIPT in EPL cases without availability of POC samples. GWNIPT detected anomalies in 24 of the 50 studied cases.

GWNIPT Result	*n* Cases	GA wks	FFE %	Size (Mb)	Pathogenicity ^ϒ^
T4	1	9	7	-	Pat
T6	1	10	4	-	Pat
T9	2	7/7 *	5/6 *	-	Pat
T14 + T20	1	7	5	-	Pat
T15	2	10/6 *	3/9 *	-	Pat
T16	2	11/7 *	3/9 *	-	Pat
T20	2	5/9 *	5/6 *	-	Pat
T21	3	9/10/9 *	4/7/17 *	-	Pat
T22	3	11/6/12 *	4/5/8 *	-	Pat
X0	2	8/9 *	8/9 *	-	Pat
T16 + dup(1)(q21.3q25.1)	1	9	5	20.0	Pat
dup(1)(p35.2q41) + dup(20)(p13q13.13)	1	8	4	183.8 + 48.8	Pat
dup(1)(p13.3q32.2)	1	-	3	99.5	Pat
dup(12)(q23.1q24.32)	1	12	6	28.6	VUS
del(6)(q24.3q27)	1	9	3	23.3	Pat

GA, Gestational Age; wks, weeks; FFE, Fetal Fraction Estimate; Pat, pathogenic. * GA and FFE of each of the cases with the same detected anomaly described in the first column. ^ϒ^ According to the guides of the American College of Medical Genetics [18,19].

## Data Availability

The original contributions presented in the study are included in the article; further inquiries can be directed to the corresponding author.
